# Prevalence of Risk Factors for Cardiovascular Disease and Their Associations with Diet and Physical Activity in Suburban Beijing, China

**DOI:** 10.2188/jea.JE20090119

**Published:** 2010-05-05

**Authors:** Lei Zhang, Li-Qiang Qin, Ai-Ping Liu, Pei-Yu Wang

**Affiliations:** 1 Department of Social Medicine and Health Education, School of Public Health, Peking University, Beijing, China; 2 Department of Nutrition and Food Hygiene, School of Radiation Medicine & Public Health, Soochow University, Suzhou, China

**Keywords:** cardiovascular disease, risk factors, associations, dietary habits, physical activity

## Abstract

**Background:**

We calculated new prevalences of risk factors for cardiovascular disease (CVD) and examined their associations with dietary habits and physical activity in a suburban area of Beijing—one of the most urbanized cities in China.

**Methods:**

In 2007, a cross-sectional survey of a representative sample of 19 003 suburban residents aged 18 to 76 years was conducted. Dietary and anthropometric data were collected by questionnaire, and blood pressure, fasting blood glucose, and serum lipids were measured.

**Results:**

The age-standardized prevalences of the CVD risk factors overweight/obesity, diabetes, hypertension, dyslipidemia, and metabolic syndrome (MS) were 31.9%, 6.1%, 33.6%, 30.3%, and 11.6%, respectively. The adjusted odd ratios (95% confidence interval [CI]) of overweight/obesity, diabetes, hypertension, dyslipidemia, and MS for participants who were physically active, as compared with those who were not physically active, were 0.67 (0.47 to 0.85), 0.87 (0.80 to 0.95), 0.92 (0.87 to 0.98), 0.89 (0.82 to 0.96), and 0.74 (0.62 to 0.89), respectively. The adjusted odds ratios (95% CI) of hypertension and MS for participants with a high intake of salt, as compared with those without a high intake of salt, were 1.72 (1.29 to 2.03) and 1.48 (1.16 to 1.77), respectively. In addition, participants who consumed a high-fat diet were more likely to be overweight/obese and dyslipidemic, whereas vegetarians had less risk of overweight/obesity, diabetes, hypertension, dyslipidemia, and MS.

**Conclusions:**

In this population of adults living in suburban Beijing, there were relatively high prevalences of the CVD risk factors overweight/obesity, diabetes, hypertension, dyslipidemia, and MS. Healthy dietary habits and physical activity may reduce the risks of these conditions.

## INTRODUCTION

Cardiovascular disease (CVD) is the leading cause of mortality worldwide. China and other economically developing countries have experienced a CVD epidemic in recent decades.^1^ Furthermore, CVD morbidity and mortality are predicted to increase in China during the next 20 years.^2^

Overweight/obesity is an important modifiable risk factor for CVD.^3^ Diabetes, hypertension, and dyslipidemia are independent risk factors for mortality from CVD and all-cause mortality.^4^^–^^6^ In recent years, the clustering of risk factors, including hyperglycemia, hypertension, hypertriglyceridemia, low high-density lipoprotein cholesterol, and overweight/obesity, has been referred to as metabolic syndrome (MS). MS is strongly associated with an increased risk of CVD, and this relation is now widely recognized.^7^^–^^9^

With economic growth and the urbanization of lifestyle and diet, suburban residents of Beijing are now experiencing a rapid increase in the prevalence and burden of CVD and other chronic diseases. To provide new data for policy planners and health education programs, we estimated the prevalences of CVD risk factors and examined their associations with dietary habits and physical activity in a population from suburban Beijing.

## METHODS

### Study population

A cross-sectional population survey of CVD, other chronic diseases, and related health behaviors was carried out using a 3-stage stratified sampling method. First, 5 towns were randomly selected in the suburbs of Beijing; these towns comprised 76 villages/street districts. Next, 30 of these 76 villages/street districts were randomly selected. Finally, a total of 20 655 people aged 18 to 76 years were randomly selected from the 30 districts and invited to participate. A total of 19 003 people (7148 men and 11 855 women) completed the survey and examination. The overall response rate was 92.0% (89.7% of men and 92.9% of women).

The study was approved by an ethics committees and other relevant regulatory bodies in Beijing, and informed consent was obtained from every participant before data collection. Participants with untreated conditions identified during the examination were referred to a primary health-care provider.

### Data collection

Data collection was conducted in a health center or community clinic in the participant’s residential area. During a study visit, trained research staff used a standardized questionnaire to collect information on the subject’s age, sex, education, medical history, family history, physical activity, alcohol use, dietary habits, and cigarette smoking.

### Blood pressure and anthropometric measurements

Blood pressure was measured 3 times with participants in a seated position, after at least 5 minutes of rest, using a calibrated mercury sphygmomanometer. One of 4 cuff sizes (pediatric, and adult regular, large, and thigh) was chosen based on the participant’s arm circumference.^10^ Participants were advised to avoid cigarette smoking, alcohol, caffeinated beverages, and exercise for at least 30 minutes before measurement. Body weight and height were measured twice during the interview. With each participant wearing light indoor clothing without shoes, weight was measured to the nearest 0.1 kg on electronic scales placed on a firm, level surface. A wall-mounted stadiometer was used to measure height, without shoes, to the nearest 0.1 cm.

### Laboratory measurements

Overnight fasting blood specimens were collected by venipuncture for measurement of serum lipids and plasma glucose. Participants who had not fasted were asked to visit centers again when their fasting time was longer than 10 hours. Blood specimens were centrifuged and plasma was stored at −80°C until laboratory assay. Plasma glucose was measured using a modified hexokinase enzymatic method (Hitachi automatic clinical analyzer, model 7060, Japan). Concentrations of total cholesterol (TC), high-density lipoprotein (HDL) cholesterol, and triglycerides (TG) were assessed enzymatically with commercially available reagents.^11^ Lipid measurements were standardized according to the guidelines of the Centers for Disease Control and Prevention—National Heart, Lung, and Blood Institute Lipid Standardization Program.^12^ The concentration of low-density lipoprotein (LDL) cholesterol was calculated by using the Friedewald equation (LDL cholesterol = TC − HDL cholesterol − TG/5) for participants with a TG level lower than 4.5 mmol/L.^13^

### Criteria for data interpretation

Body mass index (BMI) was calculated as body weight in kilograms divided by the square of the height in meters. Overweight/obesity was defined as a BMI ≥25.

Diabetes was defined as a fasting plasma glucose (FPG) level ≥7.0 mmol/L or self-reported current treatment with antidiabetic medication (insulin or oral hypoglycemic agents).^14^

Hypertension was defined as an average (calculated from 3 measurements) systolic blood pressure (SBP) ≥140 mm Hg, an average diastolic blood pressure (DBP) ≥90 mm Hg, or self-reported current treatment with antihypertensive medication for hypertension.^15^

Dyslipidemia was defined as self-reported current treatment with a cholesterol-lowering medication or meeting at least 1 of the following criteria: total cholesterol ≥5.2 mmol/L, triglycerides ≥1.7 mmol/L, HDL cholesterol <1.0 mmol/L, or LDL cholesterol ≥3.4 mmol/L.^12^

The definition and criteria for MS recommended by the China Diabetes Society (CDS) were used in this study.^16^ MS was defined as the presence of 3 or more of the following risk factors: BMI ≥25; serum triglyceride concentration ≥1.7 mmol/L or HDL cholesterol concentration <0.9 mmol/L in men or <1.0 mmol/L in women; blood pressure ≥140/90 mm Hg or treatment of hypertension; and serum glucose concentration ≥6.1 mmol/L or treatment of diabetes.

A high-salt diet was defined as an average salt intake ≥12 grams per day (as determined by using weighed food records), which exceeds the average intake of the Chinese population as indicated in the China National Nutrition and Health Survey (CNNS) in 2002.^17^ A high-sugar diet was defined as sweet and dessert intake ≥3 times/week (as determined by food frequency questionnaire). A high-fat diet was defined as an average edible oil intake ≥42 grams per day (as determined by using weighed food records), which exceeds the average intake of the Chinese population.^17^

A vegetarian was defined as a person who does not eat meat, as determined by the questionnaire.

### Statistical analysis

Data were analyzed using SAS (version 9.1, 2005, SAS Institute Inc, Cary, NC), and were standardized to the age distribution of Chinese adults in the year 2000. Descriptive statistics were computed for all variables, including means for continuous variables, frequencies for categorical variables, and standard error of the mean. Odds ratios (ORs) with 95% confidence intervals (CIs) adjusted for age, sex, smoking, drinking, and other potential confounders were calculated by multivariate logistic regression to estimate the association of CVD risk factors with dietary habits and physical activity. Differences in continuous variables were tested using the *t* test for independent samples; prevalence values for categorical variables were compared using the χ^2^ test and the trend χ^2^ test for proportions. Statistical significance was established at *P* < 0.05.

## RESULTS

The general characteristics of the study participants are shown in Table 1. The average age of the participants was 48.0 (95% CI, 46.5 to 49.6) years. The average age of men (47.7, 45.3 to 49.1) and women (48.2, 46.9 to 49.8) did not significantly differ (*P* > 0.05). Men had a higher body weight, SBP, DBP, and TG (*P* < 0.01 for all comparisons); women had a higher FPG, TC, LDL cholesterol, and HDL cholesterol (*P* < 0.05 for all comparisons).

**Table 1.** General characteristics of the study participants, expressed as means (95% CI)

Characteristics	Total (*n* = 19 003)	Men (*n* = 7148)	Women (*n* = 11 855)	*P*
Age (years)	48.0 (46.5–49.6)	47.7 (45.3–49.1)	48.2 (46.9–49.8)	>0.05
Weight (kg)	68.0 (66.7–69.4)	74.1 (72.2–75.6)	64.5 (63.3–65.9)	<0.01
BMI (kg/m^2^)	23.7 (23.2–24.3)	23.8 (23.2–24.4)	23.6 (23.1–24.2)	>0.05
SBP (mm Hg)	127.9 (125.5–130.3)	129.5 (127.0–131.7)	127.0 (124.6–129.9)	<0.01
DBP (mm Hg)	83.7 (82.1–85.4)	84.5 (82.9–86.3)	80.5 (78.8–82.2)	<0.01
FPG (mmol/l)	5.60 (5.49–5.72)	5.56 (5.45–5.67)	5.66 (5.53–5.78)	<0.01
TC (mmol/L)	4.76 (4.65–4.86)	4.74 (4.64–4.85)	4.78 (4.67–4.89)	0.02
TG (mmol/L)	1.61 (1.57–1.68)	1.67 (1.62–1.73)	1.58 (1.54–1.63)	<0.01
HDL-C (mmol/L)	1.37 (1.33–1.42)	1.34 (1.30–1.36)	1.39 (1.34–1.45)	<0.01
LDL-C (mmol/L)	2.79 (2.70–2.87)	2.76 (2.66–2.83)	2.81 (2.73–2.90)	<0.01

Abbreviations: CI, confidence interval; BMI, body mass index; SBP, systolic blood pressure; DBP, diastolic blood pressure; FPG, fasting plasma glucose; TC, total cholesterol; TG, triglycerides; HDL-C, high-density lipoprotein cholesterol; LDL-C, low-density lipoprotein cholesterol.

The age-standardized prevalences of overweight/obesity, diabetes, hypertension, dyslipidemia, and MS were 31.9%, 6.1%, 33.6%, 30.3%, and 11.6%, respectively (Table 2). The prevalences of overweight/obesity, hypertension, dyslipidemia, and MS were higher in men than in women (*P* < 0.01 for all comparisons). However, the prevalence of diabetes was higher in women than in men (*P* < 0.01). The prevalences of diabetes, hypertension, and MS increased with age among both men and women (*P* for trend <0.01 for all). In both sexes, the prevalence of overweight/obesity increased in participants younger than 50 years, and decreased in participants older than 50 years (*P* for trend, <0.01 for both). The prevalence of dyslipidemia rose with increasing age in women (*P* for trend, <0.01), and decreased with increasing age in men older than 50 years (*P* for trend <0.01).

**Table 2.** Prevalence of CVD risk factors by sex and age, expressed as percentages (SE)

Groups	Overweight/obesity	Diabetes	Hypertension	Dyslipidemia	MS
Total^a^	31.9 (0.8)	6.1 (0.3)	33.6 (1.2)	30.3 (1.0)	11.6 (0.6)
Men					
Overall^a^	32.4 (1.1)	5.6 (0.5)	35.7 (1.5)	30.6 (1.2)	12.2 (0.9)
Age, y					
18–29	27.3 (1.1)	2.2 (0.4)	7.8 (0.5)	17.1 (0.8)	2.9 (0.5)
30–39	30.6 (1.3)	2.3 (0.3)	15.9 (0.9)	27.6 (1.2)	5.1 (0.8)
40–49	34.1 (1.6)	4.3 (0.5)	30.2 (1.2)	39.8 (1.5)	11.4 (0.9)
50–59	33.8 (1.5)	6.1 (0.7)	43.4 (1.7)	32.2 (1.4)	14.1 (1.1)
60–69	29.4 (1.3)	7.7 (0.9)	52.9 (2.2)	27.1 (1.1)	16.9 (1.3)
≥70	26.5 (1.4)	8.4 (1.0)	56.3 (2.5)	26.4 (1.2)	22.6 (1.5)
Women					
Overall^a^	31.6 (1.0)	6.4 (0.4)	32.3 (1.3)	30.1 (1.1)	11.2 (0.7)
Age, y					
18–29	25.4 (0.9)	2.1 (0.3)	3.9 (0.3)	11.5 (0.7)	2.0 (0.4)
30–39	29.5 (1.1)	3.3 (0.5)	9.0 (0.7)	15.4 (0.8)	4.7 (0.9)
40–49	34.6 (1.5)	5.0 (0.5)	28.9 (1.1)	26.7 (1.2)	10.2 (1.0)
50–59	33.1 (1.5)	6.5 (0.6)	42.8 (1.7)	35.3 (1.3)	13.3 (0.9)
60–69	29.7 (1.3)	7.5 (0.8)	55.1 (2.1)	37.6 (1.3)	17.5 (1.4)
≥70	27.2 (1.6)	8.9 (1.0)	59.6 (2.6)	38.1 (1.6)	25.8 (1.6)

^a^Age-standardized prevalence rate.

Abbreviations: SE, standard error; MS, metabolic syndrome.

Table 3 shows the prevalences of CVD risk factors with respect to physical activity and dietary habits. As compared with inactive participants, those who were physically active >30 minutes/session and >3 times/week had lower prevalences of overweight/obesity (28.3% vs 34.7%), diabetes (5.0% vs 6.9%), hypertension (31.4% vs 35.2%), dyslipidemia (27.5% vs 33.1%), and MS (9.8% vs 13.2%; *P* < 0.05 for all comparisons). Participants with a high-salt diet had higher prevalences of hypertension (37.3% vs 30.7%) and MS (12.4% vs 10.3%) than did those whose salt intake was not high (*P* < 0.05 for both). The prevalences of overweight/obesity, diabetes, and MS were higher in participants with a high intake of sugar than in those whose sugar intake was not high (*P* < 0.05 for all comparisons). Those consuming a high-fat diet had higher prevalences of overweight/obesity (35.6% vs 27.9%), dyslipidemia (33.9% vs 27.2%), and MS (13.0% vs 10.2%) than did those whose fat intake was not high (*P* < 0.05 for all comparisons). Vegetarians and vegans had lower prevalences of overweight/obesity, diabetes, hypertension, dyslipidemia, and MS (*P* < 0.05 for all comparisons).

**Table 3.** Prevalence of CVD risk factors by physical activity and dietary habits, expressed as percentages (SE)

Groups	Overweight/obesity	Diabetes	Hypertension	Dyslipidemia	MS
Total^a^	31.9 (0.8)	6.1 (0.3)	33.6 (1.2)	30.3 (1.1)	11.6 (0.6)
Physical activity					
Inactive	34.7 (1.2)	6.9 (0.5)	35.2 (1.4)	33.1 (1.3)	13.2 (0.9)
Active^b^	28.3 (1.0)^d^	5.0 (0.3)^c^	31.4 (1.2)^c^	27.5 (1.2)^c^	9.8 (0.7)^d^
Dietary habits					
Salt appetite					
No	31.6 (1.1)	6.2 (0.4)	30.7 (1.2)	29.1 (1.2)	10.3 (0.5)
Yes	32.3 (1.2)	5.9 (0.4)	37.3 (1.5)^d^	31.8 (1.4)	12.4 (0.8)^c^
Sugar preference					
No	29.0 (1.1)	5.1 (0.4)	32.9 (1.3)	28.9 (1.2)	10.1 (0.6)
Yes	33.7 (1.3)^c^	6.8 (0.5)^c^	34.1 (1.4)	31.6 (1.3)	12.8 (0.7)^c^
High-fat preference					
No	27.9 (1.1)	5.8 (0.5)	33.0 (1.2)	27.2 (1.1)	10.2 (0.6)
Yes	35.6 (1.3)^d^	6.4 (0.4)	34.4 (1.3)	33.9 (1.4)^d^	13.0 (0.8)^c^
Vegetarian					
No	34.8 (1.4)	7.0 (0.5)	36.3 (1.5)	32.7 (1.3)	12.9 (0.8)
Yes	28.5 (1.1)^d^	5.0 (0.4)^d^	31.1 (1.3)^c^	28.0 (1.2)^c^	10.0 (0.7)^c^

^a^Age-standardized prevalence rate.

^b^>30 minutes/session and >3 times/week.

^c^*P* < 0.05, ^d^*P* < 0.01.

Abbreviations: SE, standard error; MS, metabolic syndrome.

**Figure 1.**
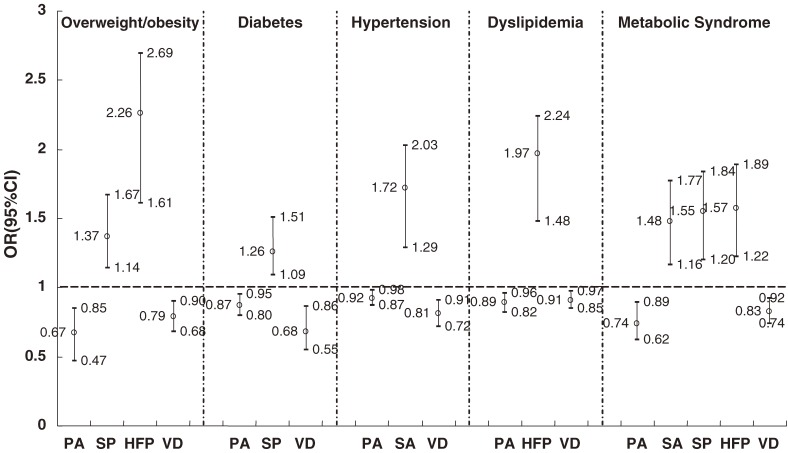
Adjusted* odds ratios of CVD risk factors, by dietary habits and physical activity, on multivariate logistic regression analysis (PA = physical activity, HS = high-salt diet, SU = high-sugar diet, HF = high-fat diet, VD = vegetarian diet). *All variables (ie, age, sex, smoking, drinking, and other potential confounders) were included in the models to calculate the adjusted odds ratios of CVD risk factors.

Adjusted ORs with 95% CIs were calculated by multivariate logistic regression to estimate the associations of CVD risk factors with dietary habits and physical activity (Figure 1). The adjusted ORs (95% CI) of overweight/obesity, diabetes, hypertension, dyslipidemia, and MS for physically active participants, as compared with those who were not physically active, were 0.67 (0.47 to 0.85), 0.87 (0.80 to 0.95), 0.92 (0.87 to 0.98), 0.89 (0.82 to 0.96), and 0.74 (0.62 to 0.89), respectively. The adjusted ORs (95% CI) of hypertension and MS for participants with a high-salt diet, as compared with those whose salt intake was not high, were 1.72 (1.29 to 2.03) and 1.48 (1.16 to 1.77), respectively. In addition, after multivariable adjustment, participants consuming a high-fat diet were more likely to be overweight/obese and dyslipidemic, and to have MS, as compared with others. Vegetarians had lower risks of overweight/obesity, diabetes, hypertension, dyslipidemia, and MS (*P* < 0.01 for all comparisons).

## DISCUSSION

Among residents of suburban Beijing aged 18 to 76 years, the prevalences of overweight/obesity, diabetes, hypertension, dyslipidemia, and MS were 31.9%, 6.1%, 33.6%, 30.3%, and 11.6%, respectively. The prevalences of overweight/obesity and diabetes in the present study were higher than in the general Chinese population,^18^ but lower than in a Beijing population.^19^ However, the prevalences of hypertension and dyslipidemia in this study were higher than those noted in general Chinese and Beijing populations, possibly because the participants of these studies lived in different areas. Beijing is the capital and one of the largest cities in China. Suburban Beijing is experiencing rapid industrialization and urbanization, which has dramatically changed individual lifestyles. Thus, the relatively high prevalences of CVD risk factors that we observed in our study are not surprising. However, it should be noted that our study was conducted in 2007 and that the high prevalences we observed were at least partly due to real increases in prevalence from 2002—when the CNNS was conducted—to 2007, when our study took place. The prevalence of MS in a Shanghai community population of 2048 people and in a Qingdao community population of 2634 people aged 20 to 74 years was 14.4% and 17.5%, respectively,^18^ which are both higher than that of the present study. Because of differences in the definitions used, sample selection, and prevalence estimation, caution is advised in directly comparing these findings.

Unhealthy behavioral factors, including high-fat, high-salt, and high-calorie diets and sedentary lifestyles, are the most salient contributors to the prevalence and mortality of CVD.^20^ The current Chinese diet, especially in urban areas, is generally high in fat (the fat energy percentage was 29.6%, in the CNNS of 2002),^17^ sodium, and energy intake, and low in fruit and vegetable intake. In addition, energy expenditure has declined among Chinese as urbanization and industrialization have progressed.^21^

Many studies have shown that the prevalence of hypertension is positively correlated with increasing salt intake.^22^^,^^23^ We also observed that high salt intake was clearly associated with hypertension, because participants with a high salt intake had high prevalences and risks of hypertension and MS (1 component of which is hypertension). Although the daily recommended salt intake for Chinese is no more than 6 grams,^24^ the estimated average salt intake is 12 grams per day,^17^ which exceeds the upper limit of the daily recommended amount. These findings suggest that the potential health benefits from limiting salt intake are of considerable importance to public health in China.

Heavy consumption of sweets and desserts such as cake and sugar-sweetened soft drinks is associated with an increased incidence of type 2 diabetes, which is also a risk factor for CVD.^25^ In people with type 2 diabetes, a low-glycemic index diet may not only be beneficial for long-term glycemic control—it may also improve other CVD risk factors.^26^ The present study found high prevalences and risks of overweight/obesity, diabetes, and MS in participants who ate a high-sugar diet.

The risks of a high-fat diet and the protection afforded by a vegetarian diet were also noted in the present study. Vegetarian and other low-fat diets generally increase intakes of carbohydrate, fiber, and several micronutrients,^27^ which are associated with reduced incidences of CVD and its risk factors, including overweight,^28^^,^^29^ type 2 diabetes,^30^^,^^31^ and plasma cholesterol.^32^^,^^33^ In a randomized intervention trial, CVD patients who followed a low-fat diet with increased intakes of fruit and vegetables, had lower CVD mortality (relative risk: 0.59, 95% CI: 0.46 to 0.74).^34^ These health effects were attributed to the fiber and phytochemicals commonly found in fruit and vegetables.^34^

There has been a progressive decline in energy expenditure among Chinese, because of physical inactivity attributable to the effects of urbanization and industrialization, which has led to more time spent watching television, playing video games, going to work by car, and using the Internet. There is convincing evidence that physical activity is related to well-known CVD risk factors such as abdominal and general obesity, diabetes, hypertension, dyslipidemia, and MS.^35^^–^^40^ In brief, high levels of physical activity reduce the age-related increases in waist circumference and weight gain,^41^ and improve lipid profile^42^ and blood pressure.^41^ Conversely, a reduction in physical activity level may aggravate the CVD risk factor profile in both sexes.^43^ One study found that for each 1-hour increase in television viewing there was a 1% to 2% increase in the prevalence of pediatric obesity in urban areas in China.^44^ In the present study, we observed inverse associations between CVD risk factors and physical activity.

### Conclusion

A large proportion of suburban adults in Beijing suffer from overweight/obesity, diabetes, hypertension, dyslipidemia, and MS—the risk factors for CVD. Economic development and the resulting changes in diet and lifestyle are the likely explanations for the high and increasing prevalence of these risk factors, which are becoming a major public health concern in rapidly urbanizing areas of China. The present and other studies have confirmed that healthy dietary habits and physical activity are helpful for preventing these risk factors. Therefore, comprehensive national strategies aimed at the prevention of risk factors for CVD are urgently needed to reduce the prevalence and societal burden of CVD in China.

## References

[1] Murray CJ , Lopez AD Mortality by cause for eight regions of the world: Global Burden of Disease Study . Lancet. 1997;349:1269–76 10.1016/S0140-6736(96)07493-4 9142060

[2] Wu Z , Yao C , Zhao D , Wu G , Wang W , Liu J , Sino-MONICA project: a collaborative study on trends and determinants in cardiovascular diseases in China, Part i: morbidity and mortality monitoring . Circulation. 2001;103:462–8 11157701 10.1161/01.cir.103.3.462

[3] Gu D , Reynolds K , Wu X , Chen J , Duan X , Reynolds RF , Prevalence of the metabolic syndrome and overweight among adults in China . Lancet. 2005;365:1398–405 10.1016/S0140-6736(05)66375-1 15836888

[4] Liu J , Hong Y , D’Agostino RB Sr , Wu Z , Wang W , Sun J , Predictive value for the Chinese population of the Framingham CHD risk assessment tool compared with the Chinese Multi-Provincial Cohort Study . JAMA. 2004;291:2591–9 10.1001/jama.291.21.2591 15173150

[5] Sjöström L , Narbro K , Sjöström CD , Karason K , Larsson B , Wedel H , Effects of bariatric surgery on mortality in Swedish obese subjects . N Engl J Med. 2007;357:741–52 10.1056/NEJMoa066254 17715408

[6] He J , Gu D , Reynolds K , Wu X , Muntner P , Zhao J , Serum total and lipoprotein cholesterol levels and awareness, treatment, and control of hypercholesterolemia in China . Circulation. 2004;110:405–11 10.1161/01.CIR.0000136583.52681.0D 15238453

[7] National Cholesterol Education Program (NCEP) Expert Panel on Detection, Evaluation, and Treatment of High Blood Cholesterol in Adults (Adult Treatment Panel III) Third report of the National Cholesterol Education Program (NCEP) Expert Panel on Detection, Evaluation, and Treatment of High Blood Cholesterol in Adults (Adult Treatment Panel III) final report . Circulation. 2002;106:3143–421 12485966

[8] Grundy SM , Cleeman JI , Daniels SR , Donato KA , Eckel RH , Franklin BA , ; American Heart Association; National Heart, Lung, and Blood Institute Diagnosis and management of the metabolic syndrome: an American Heart Association/Nation, Lung, and Blood Institute scientific statement . Circulation. 2005;112:2735–52 10.1161/CIRCULATIONAHA.105.169404 16157765

[9] Alberti KG , Zimmet P , Shaw J Metabolic syndrome-a new word-wide definition. A consensus statement form the International Diabetes Federation . Diabet Med. 2006;23:469–80 10.1111/j.1464-5491.2006.01858.x 16681555

[10] Perloff D , Grim C , Flack J , Frohlich ED , Hill M , McDonald M , Human blood pressure determination by sphygmomanometry . Circulation. 1993;88:2460–70 8222141 10.1161/01.cir.88.5.2460

[11] Allain CC , Poon LS , Chan CS , Richmond W , Fu PC Enzymatic determination of total serum cholesterol . Clin Chem. 1974;20:470–5 4818200

[12] Myers GL , Cooper GR , Winn CL , Smith SJ The Centers for Disease Control-National Heart, Lung and Blood Institute Lipid Standardization Program. An approach to accurate and precise lipid measurements . Clin Lab Med. 1989;9:105–35 2538292

[13] Friedewald WT , Levy RI , Fredrickson DS Estimation of the concentration of low-density lipoprotein cholesterol in plasma, without use of the preparative ultracentrifuge . Clin Chem. 1972;18:499–502 4337382

[14] Expert Committee on the Diagnosis and Classification of Diabetes Mellitus Report of the Expert Committee on the Diagnosis and Classification of Diabetes Mellitus . Diabetes Care. 2003;26 Suppl 1:S5–20 10.2337/diacare.26.2007.S5 12502614

[15] Chobanian AV , Bakris GL , Black HR , Cushman WC , Green LA , Izzo JL Jr , Seventh Report of the Joint National Committee on Prevention, Detection, Evaluation, and Treatment of High Blood Pressure . Hypertension. 2003;42:1206–52 10.1161/01.HYP.0000107251.49515.c2 14656957

[16] The Cooperation Group of the Chinese Medical Association Diabetes Branch Studying on the Metabolic Syndrome Suggestion for the metabolic syndrome by the Chinese Medical Association Diabetes Branch . Chin J Diabetes. 2004;12:156–61 (in Chinese)

[17] Wang L. General Report of China National Nutrition and Health Survey in 2002. Beijing: People's Health Publishing Housel; 2005 (in Chinese).

[18] National Center for Cardiovascular Diseases, China. Report on Cardiovascular Diseases in China. 1th ed. Beijing: Encyclopedia of China Publication; 2008.

[19] Liu Z. The Report of Nutrition and Health Status Survey in 2002 in Beijing. 1th ed. Beijing: Science and Technology of China Publication; 2003 (in Chinese).

[20] Liou D , Bauer KD Exploratory investigation of obesity risk and prevention in Chinese Americans . J Nutr Educ Behav. 2007;39:134–41 10.1016/j.jneb.2006.07.007 17493563

[21] Bauman A , Allman-Farinelli M , Huxley R , James WP Leisure-time physical activity alone may not be a sufficient public health approach to prevent obesity-a focus on China . Obes Rev. 2008;9 Suppl 1:119–26 10.1111/j.1467-789X.2007.00452.x 18307713

[22] Tsai PS , Ke TL , Huang CJ , Tsai JC , Chen PL , Wang SY , Prevalence and determinants of prehypertension status in the Taiwanese general population . J Hypertens. 2005;23:1355–60 10.1097/01.hjh.0000173517.68234.c3 15942457

[23] Chinese Nutrition Society. Chinese Dietary Guidelines. Lhasa: Tibet People’s Publishing House; 2008 (in Chinese).

[24] Sun Z , Zheng L , Xu C , Li J , Zhang X , Liu S , Prevalence of pre-hypertension, hypertension and associated risk factors in Mongolian and Han Chinese populations in Northeast China . Int J Cardiol. 2008;128:250–4 10.1016/j.ijcard.2007.08.127 18160149

[25] Palmer JR , Boggs DA , Krishnan S , Hu FB , Singer M , Rosenberg L Sugar-sweetened beverages and incidence of type 2 diabetes mellitus in African American Women . Arch Intern Med. 2008;168:1487–92 10.1001/archinte.168.14.1487 18663160 PMC2708080

[26] Kendall CW , Josse AR , Wong JM , Sievenpiper J , Jenkins DJ The effect of a low glycemic indexes diet on markers of cardiovascular disease risk in type 2 diabetes . J Clin Lipidol. 2008;2 Suppl 1:S138 10.1016/j.jacl.2008.08.297

[27] Turner-McGrievy GM , Barnard ND , Cohen J , Jenkins DJ , Gloede L , Green AA Changes in Nutrient Intake and Dietary Quality among Participants with Type 2 Diabetes Following a Low-Fat Vegan Diet or a Conventional Diabetes Diet for 22 Weeks . J Am Diet Assoc. 2008;108:1636–45 10.1016/j.jada.2008.07.015 18926128

[28] Barnard ND , Scialli AR , Turner-McGrievy G , Lanou AJ , Glass J The effects of a low-fat, plant-based dietary intervention on body weight, metabolism, and insulin sensitivity . Am J Med. 2005;118:991–7 10.1016/j.amjmed.2005.03.039 16164885

[29] Rosell M , Appleby P , Spencer E , Key T Weight gain over 5 years in 21,966 meat-eating, fish-eating, vegetarian, and vegan men and women in EPIC-Oxford . Int J Obes (Lond). 2006;30:1389–96 10.1038/sj.ijo.0803305 16534521

[30] Leitzmann C Vegetarian diets: what are the advantages? Forum Nutr. 2005;2:147–56 10.1159/000083787 15702597

[31] Barnard ND , Cohen J , Jenkins DJ , Turner-McGrievy G , Gloede L , Jaster B , A low-fat vegan diet improves glycemic control and cardiovascular risk factors in a randomized clinical trial in individuals with type 2 diabetes . Diabetes Care. 2006;29:1777–83 10.2337/dc06-0606 16873779

[32] Jenkins DJ , Kendall CW , Faulkner DA , Nguyen T , Kemp T , Marchie A , Assessment of the longer-term effects of a dietary portfolio of cholesterol lowering foods in hypercholesterolemia . Am J Clin Nutr. 2006;83:582–91 16522904 10.1093/ajcn.83.3.582

[33] Hu FB , Willett WC Optimal diets for prevention of coronary heart disease . JAMA. 2002;288:2569–78 10.1001/jama.288.20.2569 12444864

[34] Singh RB , Niaz MA , Ghosh S Effect on central obesity and associated disturbances of low-energy, fruit- and vegetable-enriched prudent diet in North Indians . Postgrad Med J. 1994;70:895–900 10.1136/pgmj.70.830.895 7870637 PMC2398005

[35] Fagard RH Exercise is good for your blood pressure: effects of endurance training and resistance training . Clin Exp Pharmacol Physiol. 2006;33:853–6 10.1111/j.1440-1681.2006.04453.x 16922820

[36] Kodama S , Tanaka S , Saito K , Shu M , Sone Y , Onitake F , Effect of aerobic exercise training on serum levels of high-density lipoprotein cholesterol: a meta-analysis . Arch Intern Med. 2007;167:999–1008 10.1001/archinte.167.10.999 17533202

[37] Slentz CA , Aiken LB , Houmard JA , Bales CW , Johnson JL , Tanner CJ , Inactivity, exercise, and visceral fat. STRRIDE: a randomized, controlled study of exercise intensity and amount . J Appl Physiol. 2005;99:1613–8 10.1152/japplphysiol.00124.2005 16002776

[38] Ford ES , Kohl HW 3rd , Mokdad AH , Ajani UA Sedentary behavior, physical activity, and the metabolic syndrome among US adults . Obes Res. 2005;13:608–14 10.1038/oby.2005.65 15833947

[39] Aaldahl M , von Huth Smith L , Pisinger C , Toft UN , Glümer C , Borch-Johnsen K , Five-year change in physical activity is associated with changes in cardiovascular disease risk factors: The Inter 99 Study . Prev Med. 2009 [Epub ahead of print]. 10.1016/j.ypmed.2009.01.015 19463487

[40] Aadahl M , Kjaer M , Jørgensen T Influence of time spent on TV viewing and vigorous intensity physical activity on cardiovascular biomarkers. The Inter 99 study . Eur J Cardiovasc Prev Rehabil. 2007;14:660–5 10.1097/HJR.0b013e3280c284c5 17925625

[41] Balkau B , Vierron E , Vernay M , Born C , Arondel D , Petrella A , The impact of 3-year changes in lifestyle habits on metabolic syndrome parameters: the D.E.S.I.R study . Eur J Cardiovasc Prev Rehabil. 2006;13:334–40 10.1097/00149831-200606000-00007 16926661 PMC4764669

[42] Sternfeld B , Wang H , Quesenberry CP Jr , Abrams B , Everson-Rose SA , Greendale GA , Physical activity and changes in weight and waist circumference in midlife women: findings from the Study of Women’s Health Across the Nation . Am J Epidemiol. 2004;160:912–22 10.1093/aje/kwh299 15496544

[43] Ekelund U , Franks PW , Sharp S , Brage S , Wareham NJ Increase in physical activity energy expenditure is associated with reduced metabolic risk independent of change in fatness and fitness . Diabetes Care. 2007;30:2101–6 10.2337/dc07-0719 17536069

[44] Ma GS , Li YP , Hu XQ , Ma WJ , Wu J Effect of television viewing on pediatric obesity . Biomed Environ Sci. 2002;15:291–7 12642985

